# Effect of dietary supplementation of prebiotic, probiotic, and synbiotic on growth performance and carcass characteristics of broiler chickens

**DOI:** 10.14202/vetworld.2016.313-319

**Published:** 2016-03-25

**Authors:** Nihar Ranjan Sarangi, L. K. Babu, A. Kumar, C. R. Pradhan, P. K. Pati, J. P. Mishra

**Affiliations:** 1 Livestock Production and Management Section, ICAR-National Dairy Research Institute, Karnal, Haryana, India; 2 Department of Livestock Production and Management, College of Veterinary Science and Animal Husbandry, Orissa University of Agriculture and Technology, Bhubaneswar, Odisha, India; 3 Directorate of Research on Women in Agriculture (ICAR-DRWA), Bhubaneswar, Odisha, India; 4 Department of Livestock Products Technology, College of Veterinary Science and Animal Husbandry, Orissa University of Agriculture and Technology, Bhubaneswar, Odisha, India; 5 Department of Livestock Products Technology, West Bengal University of Animal and Fishery Sciences, Kolkata, West Bengal, India

**Keywords:** caracass characteristics, growth performance, prebiotic, probiotic, synbiotic, Vencobb broilers

## Abstract

**Aim:**

The aim was to investigate the effects of dietary supplementations of prebiotic, probiotic, and synbiotic on growth performance and carcass characteristics of broiler chickens.

**Materials and Methods:**

A total of 360 1-day-old Vencobb broiler chickens of either sex were randomly assigned to four dietary treatments each consisting of three replicates and each replicate having 30 birds for 6 weeks. The dietary treatments were (1) control group with basal diet, (2) basal diet supplemented with prebiotic (at 400 g/tonne of starter as well as finisher ration), (3) basal diet supplemented with probiotic (at 100 g/tonne of starter ration and 50 g/tonne of finisher ration), and (4) basal diet supplemented with synbiotic(at 500 g/tonne of starter as well as finisher ration). The birds were provided with *ad-libitum* feed and drinking water during the entire experimental period.

**Results:**

The highest body weight observed in asynbiotic group, which was non-significantly (p>0.05) higher than thecontrol group. Prebiotic and probiotic groups showed lower body weight than synbiotic and control groups. A total feed intake did not show any significant (p>0.05) difference between experimental groups. There were no significant (p>0.05) differences in feed conversion ratio of broiler chickens in prebiotic, probiotic, and synbiotic groups as compared with control group. There was no significant (p>0.05) difference in the carcass traits with respect to dressing percentage, carcass percentage, heart weight, liver weight and gizzard weight, wing percentage, breast percentage, back percentage, thigh percentage, and drumstick percentage in Cobb broilers under study.

**Conclusion:**

The growth performance and percentage of carcass yield did not show any significant increase by the dietary inclusion of prebiotic, probiotic, and synbiotic compared with unsupplemented control in a commercial broiler chicken.

## Introduction

Poultry serves as one of the means of satisfying the increased demand for animal protein. Presently, chicken meat is on demand as a cheap source of protein with low cholesterol value. Therefore, adaptation of broiler farming is increasing day by day by farmers. As 70% of total cost of production is contributed by feed only, improvement of feed conversion ratio (FCR) will significantly enhance the margin of profit. Antibiotics have long been used as growth promoters. In recent years, due to theresidual effect of antibiotics on human health, the use of many antibiotics in food production is banned or going to be banned. The occurrence of cross resistance of antibiotic growth promoters with the human medicines has become an important issue at present. Moreover, the growing concern arising among the people about food safety, environmental contamination, and general health issues due to thepresence of residual antibiotics in poultry meat has driven a way to find out a solution to the use of antibiotic growth promoter. Considering these facts in mind the feeding of other non-antibiotic growth promoters such as prebiotics, probiotics, and synbiotics finds a potential substitute for antibiotics.

To promote growth, protect well-being and maximize the genetic prospective of modern broiler [1] and layer hybrids [2] growth promoter feed additives have been included in poultry diets. A prebiotic is defined as non-digestible food ingredients that beneficially affect the host, selectively stimulating the growth or activity, or both, of one or a limited number of bacteria in the colon [3]. It has been shown that prebiotics encourages the growth of endogenous microbial population groups such as *Bifidobacteria* and *Lactobacilli* which are particularly stimulated, and these bacteria species are considered as beneficial to animal health [4]. Furthermore, dietary supplementation of a fructooligosaccharide (0.3% dose) or oligochitosan (0.1% dose) as prebiotic, showed growth-promoting effects similar to antibiotic treatments based on flavomycin [5] or aureomycin [6].

Probioticsare “live microorganisms which when administered in adequate amount confer a health benefit on the host” [7,8]. Several studies reported that probiotics have beneficial effects on growth performance [9]. In broiler nutrition, probiotic species belonging to *Lactobacillus*, *Streptococcus*, *Bacillus*, *Bifidobacterium*, *Enterococcus*, *Aspergillus*, *Candida*, and *Saccharomyces* have a beneficial effect on broiler performance [10], modulation of intestinal microflora and pathogen inhibition [11], and promoting microbiological meat quality of broilers [12]. The mode of action of probiotics in poultry includes maintaining normal intestinal microflora by competitive exclusion antagonism, lowering the pH through acid fermentation, competing for mucosal attachment and nutrients, producing bacteriocins, stimulating the immune system associated with the gut, increasing production of short-chain fatty acids [13].

Synbiotic is a combination of probiotics and prebiotics [14]. This product could improve the survival of the probiotic organism because its specific substrate is available for fermentation. This could result in advantages to the host through the availability of the live microorganism. The combination of a pre- and probiotic in one product has been shown to confer benefits beyond those of either on its own. A way of potentiating the efficacy of probiotic preparations may be the combination of both prebiotics and probiotics as synbiotics that beneficially affects the host by improving the survival and implantation of live microbial dietary supplements in the gastrointestinal tract.

To assess the dietary effect of prebiotic, probiotic, and synbiotic for different purposes and different age groups of poultry birds, the present study was undertaken to study the effect of dietary supplementation of prebiotic, probiotic, and synbiotic on growth, feed consumption, FCR, mortality and carcass characteristics of broiler chickens.

## Materials and Methods

### Ethical approval

The experiment was carried out according to the National Regulations on Animal Welfare and Institutional Animal Ethics Committee.

### Place of work

The experiment was carried out in the Department of Livestock Production and Management, College of Veterinary Science and Animal Husbandry, Bhubaneswar and the Directorate of Research on Women in Agriculture (DRWA), Bhubaneswar.

### Feeding and management

A total of 360 1-day-old Vencobb broiler chicks of either sex were procured from the Eastern Hatcheries Pvt. Ltd., Bhubaneswar. The average maximum and minimum ambient temperature during the 6-week of experimental period ranged from 37.2°C to 41.3°C (average 39.25°C) and 21.7-24.8°C (average 23.8°C), respectively. There were 12 pens, each having a floor area of 40 sq. feet, i.e., 8 feet × 5 feet. The chicks were wing banded, weighed and randomly distributed into four dietary treatment groups. Each group was again divided into three replicates having 30 chicks in each replicate pen (Table-1). Fresh rice husk was used as litter material. The chicks were brooded using incandescent electric bulbs. During brooding, theadequate light of 24 h and ventilation were facilitated. Cleaned and disinfected feeders and waterers were provided in the pen as per the requirements of the birds. Fresh clean water was provided twice daily in clean waterers inside the pen. Feed and water were provided *ad libitum*. All the chicks were vaccinated against Ranikhet disease (B1 Strain, Ventri^®^) on 7^th^ and 21^st^ day of age and Infectious Bursal Disease (Live Vaccine Intermediate Standard Strain Ventri^®^) on 14^th^ and 35^th^ day of age. Routine managemental practices were adopted for all treatment groups as per standard practices.

**Table-1:** Details of experimental diets.

Treatment no.	Treatments	Number of chicks
T_1_	Basal diet (control)	90
T_2_	Basal diet+prebiotic	90
T_3_	Basal diet+probiotic	90
T_4_	Basal diet+synbiotic	90

The starter and finisher rations prepared in DRWA, Bhubaneswar were fed to chicks during the experimental period. The dietary treatments were (1) control, (2) basal diet supplemented with prebiotic (400 g per tonne of starter as well as finisher diets), (3) basal diet supplemented with probiotic (100 and 50 g per tonne of starter and finisher diets, respectively), and (4) basal diet supplemented with synbiotic (500 g per tonne of starter as well as finisher diet). The composition and dose rate of prebiotic, probiotic, and synbiotic used in the diet have been presented in Table-2.

**Table-2:** Composition of prebiotic/probiotic/synbiotic used in the diet.

Items	Prebiotic	Probiotic	Synbiotic
Composition	MOS	Each gram contains 10^9^ CFU of *L. bulgaricus, L. plantarum, S. faecium, B. bifidus,* and *S. cerevisiae*	Prebiotic: MOS (naturally derived from extracts of yeast cell walls) 14-16%. Probiotic cultures: 100 billion CFU/kg *L*. *bulgaricus, L. plantarum, S. faecium, B. bifidus*, and *S. cerevisiae*
Dose rate	400 g/tonne of starter as well as finisher ration	100 g/tonne of starterration and 50 g/tonne of finisher ration	500 g/tonne of starter as well as finisher ration

MOS=Mannan oligosaccharide, *L. bulgaricus=Lactobacillus bulgaricus, L. plantarum=Lactobacillus plantarum, S. faecium=Streptococcus faecium, B. bifidus=Bifidobacterium bifidus, S. cerevisiae=Saccharomyces cerevisiae*

The chicks were fed with starter ration up to 21 days and finisher ration from 22 to 42 days of age as per BIS (1992) recommendations. The chicks under treatment were provided with dietary supplemented ration from day old to the 42^nd^ day of age. During the period of study (0-6 weeks), all the birds were provided with starter diet (with 3005 kcal of metabolizable energy [ME]/kg of ration and 22.37% crude protein [CP]) from 0 to 3 weeks of age and finisher diet (with 3120 kcal of ME/kg of ration and 20.21% CP) from 4 to 6 weeks of age with *ad libitum* provision of water. The ingredients and nutrient composition of the feed for broilers have been presented in Table-3.

**Table-3:** Formula composition of broiler basal ration.

Ingredients (%)	Starter (0-3 weeks)	Finisher (4-6 weeks)
Maize	54.990	58.250
Deoiled rice bran	0.500	2.000
Vegetable oil (rice bran oil)	2.250	3.750
Deoiled soya meal	38.000	32.000
Choline chloride (50%)	0.120	0.150
Salt	0.250	0.250
Sodium bicarbonate	0.200	0.200
Calcite powder (Ca=34%)	1.400	1.250
Dicalcium phosphate	1.600	1.560
ABDK vitamin	0.025	0.025
Coccistat (CMP1)	0.100	-
L-Lysine	0.090	0.070
DL-Methionine	0.230	0.200
Coccidiostat (Maduramycin)	-	0.050
B-complex	0.025	0.025
Biobantox	0.100	0.100
Mineral mixture*	0.120	0.120
Total	100.00	100.00
Calculated value		
ME (kcal/kg)	3005	3120
CP %	22.37	20.21
CF %	4.00	3.94
Lysine (%)	1.328	1.153
Methionine (%)	0.570	0.554
Calcium (%)	1.00	0.92
Phosphorus (%)	0.45	0.41

* TraceMin CB (Venky’s India Private Limited, Pune).

Each 1 kg TraceMin-CB contains: Manganese=90 g, Zinc=80 g, Iron=90.0 g, Copper=15.0 g, Iodine=2.0 g, Selenium=300 mg

### Weight gain, feed consumption, FCR

The body weights of individual birds were recorded at weekly interval, and average body weight gain was calculated. Feed consumption of birds of each replicate was recorded at weekly intervals and feed consumption per bird per week was calculated. Daily mortality was recorded and due importance was given to mortality while calculating feed consumption and FCR.

### Carcass characteristics

At the end of 6^th^ week of age, three birds from each replicate were taken randomly for therecording of carcass characteristics. Birds were dressed, eviscerated and the dressed, eviscerated ready-to-cook and cut up yields were estimated as per Falaki *et al*. [4].

### Statistical analysis

The data obtained in this study were analyzed statistically in SPSS software (version 16.0) as per the methods outlined by Snedecor and Cochran [15]. The significance between the treatment groupswas analyzed by one-way ANOVA test. p value statistical significance was declared at 1% and 5%.

## Results and Discussion

### Body weight and body weight gain

**Figure-1:**
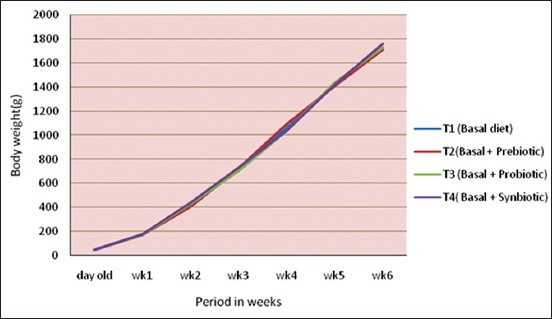
Weekly mean body weight (g) of chicks under different treatments.

**Figure-2:**
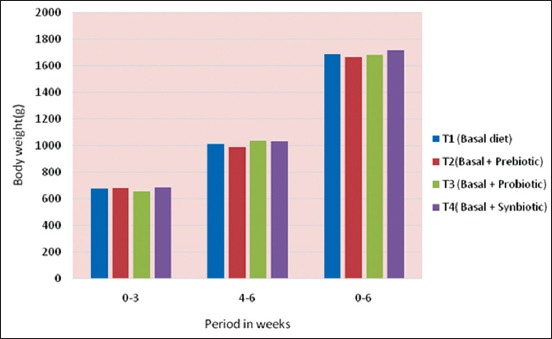
Cummulative body weight gain (g) of chicks under different treatments.

The mean day old body weight, weekly mean body weight and cumulative body weight gain of broiler chicken are presented in Table-4 and Figures-1 and 2.

**Table-4:** Effect of dietary prebiotic, probiotic, and synbiotic on growth performance of broiler chickens.

Item	Control (T_1_) n=90	Prebiotic (T_2_) n=90	Probiotic (T_3_) n=90	Synbiotic (T_3_) n=90	p value
Body weight (g)					
0 day	44.98±0.44	44.33±0.23	45.23±0.68	44.91±0.63	0.790
14 day	403.98^a^±1.70	404.79^a^±5.30	422.43^ab^±4.46	437.45^b^±3.83	0.003**
21 day	718.89±7.22	724.63±5.42	698.86±8.98	730.13±11.42	0.244
42 day	1730.04±10.27	1711.76±24.81	1726.30±25.46	1761.88±20.84	0.599
Weight gain (g)					
0-3 weeks	673.91±8.92	680.30±6.92	653.62±10.52	685.22±14.69	0.243
4-6 weeks	1011.15±5.63	987.13±27.46	1034.44±15.31	1031.75±11.83	0.251
0-6 weeks	1685.07±12.86	1667.43±30.53	1681.06±30.53	1716.97±26.27	0.606
Feed intake (g/bird)					
0-3 weeks	1083.46±15.23	1107.49±31.67	1051.54±25.57	1111.99±71.63	0.732
4-6 weeks	1847.75±32.13	1852.44±61.43	1847.51±60.90	1957.77±16.01	0.326
0-6 weeks	2931.21±17.74	2959.93±64.58	2899.05±76.91	3069.77±55.66	0.258
FCR					
0-3 weeks	1.61±0.04	1.63±0.03	1.63±0.03	1.63±0.14	0.997
4-6 weeks	1.83^ab^±0.03	1.88^ab^±0.01	1.78^a^±0.04	1.90^b^±0.01	0.044*
0-6 weeks	1.74±0.01	1.77±0.01	1.72±0.02	1.79±0.06	0.460
Mortality %	2.22	3.33	2.22	3.33	-

Number of samples-90, means bearing different superscripts in the same row differ significantly

** p<0.01,

* p<0.05).

FCR=Feed conversion ratio

The day old body weight of broiler chicks under different treatment ranged from 44.33±0.23 to 45.23±0.68 g. As the age increased, the live weight of the chicks under different treatment increased steadily up to 6 weeks of age reaching the lowest body weight in T_2_, i.e. prebiotic diet group and highest body weight in T_4_, i.e., synbiotic diet group.

The cumulative body weight gain showed an increasing trend reaching the highest value in T_4_, i.e., thesynbiotic group as against in T_2_, i.e., prebiotic group up to 6 weeks of age. There was no significant difference in the cumulative body weight gain of broilers between different treatments from 1^st^ to 6^th^ weeks of age excepting the 2^nd^ week where there was a significant difference in the body weight gain in T_4_, i.e., thesynbiotic group as compared to T_1_, i.e. control group and T_2_, i.e., prebiotic group. However, differences in the cumulative body weight gain of broilers between T_3_ and T_4_ and between T_1_, T_2_, and T_3_ groups were found to be non-significant. The cumulative live weight gain increased during second 3 weeks of age (4-6 weeks of age) as during first 3 weeks (0-3 weeks) of age. This could be due to the proper direct fed microbial (DFM) supplementation promoting favorable condition in the intestine for the colonization of beneficial microflora, which in turn facilitated better growth performance of broiler chicks [16]. However, the results of thepresent study were found contrary to Lee *et al*. [17] who hadshown that body weight gain was not influencedby the addition of dietary DFM in the broilerdiets.

The cumulative gain a bird was higher in T_4_ than T_1_, although the differences were statistically non-significant during 0-6 weeks of age and it was not in accordance withthe findings of Torres-Rodriguez *et al*. [18]. The present findings were in agreement with Awad *et al*. [19] who reported that addition of probiotic to broilers did not show any significant effect on body weight compared with control group. Similarly, Appelt *et al*. [20] found that addition of prebiotics had no significant effect on weight gain of broiler chickens. In contrast, Nikpiran *et al*. [21] observed that diets containing prebiotics and probiotics increased body weight of broilers significantly in comparison to control group and Awad *et al*. [19] found that inclusion of synbiotic increased daily weight gain of broilers significantly whereas addition of probiotic had no significant effect. This irregularity in the effectiveness of prebiotics might be due to the different factors such as environmental effects, irregularity in management, diet, type of additive used, and amount of additive Nikpiran *et al*. [21].

### Feed consumption and FCR

**Figure-3:**
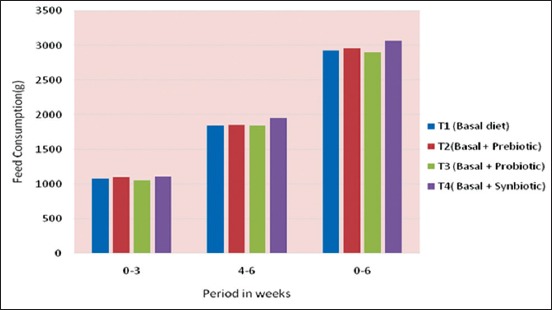
Cummulative feed consumption (g) per chick under different treatment.

The cumulative feed consumption per chick under different treatments is presented in Table-4 and Figure-3. The cumulative feed consumption per chick was higher for T_4_ (synbiotic group) as compared to T_1_ (control) during the period from 0 to 3 weeks and T_4_ group as compared to T_3_ (probiotic group) during 4-6 weeks of age. The impact of dietary supplementation of prebiotic, probiotic, and synbiotic on cumulative feed consumption during anentire period of experiment, i.e., 0-6 weeks of age was found to be non-significant (p>0.05).

The present finding was in agreement with Salma *et al*. [22] who found that feed intake of broilers did not differ significantly by dietary inclusion of probiotics. Similar results were also obtained by Jung *et al*. [23] who found that addition of prebiotic and probiotic did not have any significant effect on feed intake of broiler chickens. However, the present observation was found not in concurrence with the result of Salianeh *et al*. [24] who reported that dietary inclusion of prebiotic significantly decreased feed intake in broiler chickens as compared to control group, whereas addition of probiotic did not have the same effect as prebiotic.

**Figure-4:**
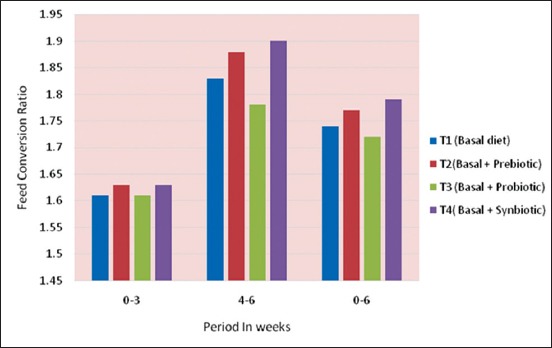
Cummulative FCR of Chicks under different treatments.

The cumulative FCR of broiler chicken is presented in Table-4 and Figure-4. The cumulative FCR of the chicks under different treatments revealed that there was a gradual increasing trend observed with age. A non-significant difference in the FCR during 0-6 weeks of age might be ascribed to similar efficiency in different treatments. During 0-3 weeksperiod, the FCR of broiler chicken ranged from 1.61 to 1.63 and did not differ significantly between treatments.

The cumulative feed conversion of the broilers during 4-6 weeks was significantly (p<0.05) higher in T_3_, the probiotic group as compared to T_4_, synbiotic group but the differences between prebiotic, probiotic and control was non-significant. Similarly, the FCR during 22-42 days did not differ significantly between prebiotic and synbiotic group compared to control. The present finding was in agreement with Talebi *et al*. [25] who reported that addition of probiotic to broiler chicken diets decreased FCR significantly. On the contrary Awad *et al*. [19] reported that dietary supplementation of synbiotic significantly decreased FCR while addition of probiotic had no significant effect. Dizaji *et al*. [26] also reported that between days 29 and 42, FCR in synbiotic and acidifier groups were significantly higher than control group. The discrepancy observed in the present study might be due to thedifference in breed and climatic condition that were provided to the chicken.

The FCR did not differ significantly between treatments during 0-6 weeks of age. The FCR during 0-42 days ranged from 1.78 (probiotic group) to 1.79 (synbiotic group) and the difference was nominal. The present FCR observedwas better than the value of 1.853-1.988 as reported by Dizaji *et al*. [26] in Ross 308 broilers. The present findings were in agreement with Mokhrati *et al*.[27] who studied the efficiency of different growth promoters and reported no significant difference between treatments in body weight gain but all of them had abeneficial effect as compared to control. Lowest FCR was observed in theprobiotic group as compared to control group and caused more efficient utilization of feed. The present findings were not in agreement with Mutus *et al*. [28] and Vargas-Rodriguez *et al*. [29] who reported that probiotic supplementationin broiler diets did not increase feed intake, weight gain, and FCR throughout the study.

### Mortality

The mortality of chicks under different treatments is presented in Table-4. The overall mortality (0-6 weeks) during the experimental period was low in thecontrol group and probiotic group as compared to prebiotic group and synbiotic group. The variation in mortality among different treatments might be due to theseasonal influence of summer season and cannot be ascribed as treatment effect. The mortality observed in the present study agreed with the report of Awad *et al*. [19] who reported lower mortality rate for probiotic supplemented group (3%) than the synbiotic supplemented group and control group (3.5%) in Ross 308 commercial broilers.

### Carcass characteristics

The carcass characteristic of broiler chicken under different treatments is presented in Table-5. The dressing percent of thecarcasswere ranged from 77.81% to 80.18%. The present findings were higher than the report of Narasimha *et al*. [30] who reported dressing yield (%) ranging from 63.67% to 66.67% in Cobb commercial broiler at 42 days of age. The carcass yield (%) in the present study ranged from 73.77% to 76.04% after 42 days of age which was more than the value observed by Abdel-Raheemand Abd-Allah [31] who reported 64.45 to 70.68% in Avian -48 broilers of 42 days of age. There was no significant difference observed in the carcass traits with respect to dressing percentage, carcass percentage, heart weight, liver weight and gizzard weight in Cobb broilers under study. The present findings were in agreement with the report of Sahin *et al*. [32] and Chumpawadee *et al*. [33] who reported that the prebiotic, probiotic, and synbiotic had no significantly (p>0.05) positive effect on carcass yields of quails and broilers. The present findings were not in agreement with Abdel-Raheemand Abd-Allah [31] who reported a significant increase (p<0.05) in the carcass weight and dressing percentage in synbiotic supplemented broilers compared with either prebiotic or probiotic alone supplemented group in Avian 48 broilers. Furthermore, Awad *et al*. [18] reported that the synbiotic supplemented group had a greater (p<0.05) carcass percentage as compared to the control group and probiotic supplementedgroup but the differences between control group and probiotic supplemented group were non-significant. The neck percentage was significantly higher in thesynbiotic group compared to prebiotic, but the differences between prebiotic, probiotic and control groups were found to be non-significant. The mean cut-off parts such as neck, wing, breast, back, thigh and drumstick expressed as percentage of eviscerated weight were 7.30%, 10.87%, 33.83%, 18.02%, 16.49% and 13.02%, respectively.

**Table-5:** Effect of dietary prebiotic, probiotic, and synbiotic on carcass characteristics of broiler chickens.

Particulars	T_1_ (basal diet)	T_2_ (basal+prebiotic)	T_3_ (basal+probiotic)	T_4_ (basal+synbiotic)	Remark
Live weight (g)	2288.6±48.13	2213.5±101.66	2302.0±109.54	2322.0±69.53	NS
Dressed weight (g)	1822.0±44.56	1767.0±70.30	11793.80±102.02	1861.60±54.35	NS
Eviscerated weight (g)	1721.5±41.36	1677.75±65.81	1701.20±99.75	1765.40±51.02	NS
Dressing %	79.56±0.51	79.93±0.48	77.81±1.26	80.18±0.57	NS
Carcass %	75.17±0.38	75.90±0.47	73.77±1.26	76.04±0.55	NS
Heart weight (g)	10.75±0.19	8.75±0.49	12.60±1.47	11.60±0.87	NS
Liver weight (g)	47.00±2.95	40.50±3.54	43.40±2.29	40.80±1.02	NS
Gizzard weight (g)	43.00±1.38	37.75±3.29	37.20±3.93	43.60±3.40	NS
Neck weight (g)	122.00^ab^±1.14	122.25^ab^±5.39	116.80^a^±5.95	139.20^b^±4.02	*
Wing weight (g)	184.00±1.58	183.50±7.81	190.40±9.92	187.80±6.13	NS
Breast weight (g)	565.25±22.28	578.50±27.46	586.80±34.52	592.60±17.28	NS
Back weight (g)	319.00±9.49	295.00±12.59	295.80±22.09	330.40±26.30	NS
Thigh weight (g)	294.75±5.49	274.50±8.59	278.20±27.09	285.80±11.20	NS
Drumstick weight (g)	227.00±5.90	218.00±7.38	221.60±10.35	225.40±2.25	NS

Means bearing different superscripts in the same row differ significantly.

* Significant (P<0.05),

**Highly significant (p<0.01). NS=Non-significant (p>0.05)

## Conclusion

A biological experiment conducted did not show any significant increase in the body weight, body weight gain, feed consumption, FCR, mortality and percentage of carcass yield by the dietary inclusion of prebiotic, probiotic, and synbiotic compared with unsupplemented control in commercial broiler chicken. The probiotic and synbiotic treatment decreased the feed: Gain ratios and probiotic treatment had decreased mortality percentage as compared to synbiotics. Therefore, these products might be promising alternatives for antibiotic growth promoters, as pressure to eliminate antibiotic growth promoters in animal feed increases.

## Authors’ Contributions

The present work was carried out during NRS’s M.V.Sc thesis program and it was an original research work. LKB, AK, CRP and PKP conceptualized the aim of the study, designed, planned and supervised the experiment and corrected the manuscript. Collection of samples, execution of the experimental study, collation and analysis of data, interpretation of the results and drafting the manuscript was done by NRS. JPM helped in analysis, drafting and revision of the manuscript. All authors read and approved the final manuscript.
